# Leptin regulates OPG and RANKL expression in Gingival Fibroblasts and Tissues of Chronic Periodontitis Patients

**DOI:** 10.7150/ijms.56151

**Published:** 2021-04-22

**Authors:** Yiting Guo, Chunjiao Xu, Xiaoshan Wu, Wenrui Zhang, Yumei Sun, Alisha Shrestha

**Affiliations:** 1 Central of Stomatology, Xiangya Hospital of Central South University, Changsha, China.; 2 Stomatological Hospital of Xiamen Medical College, Xiamen, China.

**Keywords:** chronic periodontitis, leptin, OPG, RANKL, gingival tissue, gingival fibroblasts

## Abstract

**Objective:** Chronic periodontitis is a bone-destructive disease affecting periodontal support structures. Although leptin has a protective effect against periodontitis, the underlying mechanism remains unclear. Therefore, this study aimed to investigate the possible role of leptin by examining its relationship with OPG and RANKL in human gingival tissues obtained from patients with chronic periodontitis.

**Method:** Twenty-two patients with chronic periodontitis were enrolled (10 with moderate periodontitis and 12 with severe periodontitis) in the experimental group, and 12 healthy individuals were enrolled in the control group. Gingival tissue samples were collected, and the protein levels and localization of leptin, OPG, and RANKL were studied using immunohistochemistry (IHC). The staining intensities of leptin, OPG, and RANKL were correlated with the periodontal clinical index. Moreover, real-time quantitative PCR (RT-qPCR) was used to determine *OPG* and *RANKL* mRNA levels in gingival fibroblasts stimulated with gradient concentrations of leptin protein *in vitro*.

**Result:** Leptin, OPG, and RANKL were located in the cytoplasm of gingival epithelial cells and the connective tissue. Leptin was widely and significantly expressed in the control group, whereas it was lightly stained in the severe group. RANKL was lightly stained in the control group, whereas it was widely and significantly expressed in the severe group. The control and the moderate groups had similar OPG levels, which were significantly higher than that in the severe group. Leptin was positively correlated with OPG(r = 0.905, *p* < 0.01) and negatively correlated with RANKL (r = -0.635, *p* < 0.01). *In vitro* low concentrations of leptin led to an increased *OPG*/*RANKL* mRNA ratio, whereas the opposite effect was observed at high concentrations.

**Conclusion:** Leptin can regulate OPG and RANKL expression in gingival fibroblasts and may thus play a role in the development of chronic periodontitis by modulating the *OPG/RANKL* ratio.

## Introduction

Chronic periodontitis is an inflammatory and bone-destructive disease affecting periodontal support structures. Chronic periodontitis patients often display alveolar bone resorption that can lead to gomphiasis 1. Notably, bone resorption is the leading cause of tooth loss in adults. Leptin is a 167 amino-acid protein that is encoded by the leptin gene 2. It exerts its effect by binding to its receptor, which is widely expressed in the central nervous system and peripheral tissues 3. In recent years, increasing attention has been focused on studying the role of leptin in bone metabolism. Some studies have shown that leptin may cause either osteogenesis or resorption, depending on the target tissue. Specifically, its action in the central nervous system ultimately leads to production of signals promoting bone destruction, whereas activation of targets in the peripheral tissues promotes bone formation. RANKL binds to the receptor activator activator of NF-κB (RANK), a membrane receptor mainly produced in osteoclasts and their progenitor cells. This stimulates both differentiation of precursor cells into osteoclasts and the activity of the mature osteoclasts. OPG plays a bone-protective role because it competes with RANK for RANKL binding 4. However, the relationship between leptin and OPG/RANKL in gingival tissue and human gingival fibroblasts (HGFs) has not been investigated.

Few studies have investigated the relationship between leptin and chronic periodontitis. Of note, concentrations of leptin in the saliva and the gingival crevicular fluid of chronic periodontitis patients were significantly lower than those in healthy individuals 5,6. Another study showed that leptin levels in gingival tissues decrease as the periodontal pocket deepens 7. Together, these studies indicate that leptin may be related to the development of chronic periodontitis; however, the underlying mechanism remains unclear.

This study aimed to investigate the expression of leptin, OPG, and RANKL in human gingival tissues to elucidate the role of leptin in chronic periodontitis. We analyzed the correlation between the expression of leptin and that of the other two proteins. HGFs were cultured and subjected to treatment with various concentrations of leptin to investigate the effect of leptin on OPG and RANKL expression. Importantly, the mechanism of action of leptin in the development of chronic periodontitis was also explored, which may help us to better understand, and therefore treat, chronic periodontitis in the future.

## Materials and methods

### Subjects

Twenty-two patients with chronic periodontitis from the Stomatology Center of Xiangya Hospital of Central South University were recruited for this study from March to June 2019. Among them, 10 were diagnosed with moderate chronic periodontitis (moderate group, clinical attachment loss (CAL) 3-4 mm, probing depth (PD) 5~7 mm), and 12 with severe chronic periodontitis (severe group, CAL ≥ 5 mm, PD ≥ 7 mm) 8. The control group consisted of 12 age- and sex-matched healthy subjects. The comparison of basic clinical data is shown in Table 1. Inclusion criteria included the absence of history of systemic or infectious diseases, and the absence of smoking or chronic alcohol abuse. Subjects did not receive periodontal treatment within 6 months of beginning this study; they also did not take antibiotics, immunosuppressants, or other drugs within 3 months prior to this study. Exclusion criteria included obesity, pregnancy or lactation and previous orthodontic treatment.

For the immunostaining study, gingival tissues of patients were obtained from periodontal surgical resection, whereas those of the controls were collected from gingiva removed during the extraction of the third molar. The age and sex of the subjects in the experimental and control groups were matched.

For the *in vitro* experiments, we used cells isolated from gingival tissues removed during the extraction of the third molar. The three donors used for these experiments were 15-30 years old and did not have periodontal or apical diseases.

Informed consent was obtained from all subjects and this trial was approved by the Ethics Committee of Xiangya Hospital of Central South University (Number: 2019030082).

### Reagents

Leptin Rabbit Anti-Human Polyclonal Antibody (CSB-PA009805, CUSABIO, China), OPG Rabbit Anti-Human Polyclonal Antibody (CSB-PA003597, CUSABIO, China), RANKL Rabbit Anti-Human Polyclonal Antibody (CSB-PA023986LA01HU, CUSABIO, China), DAB (Servicebio, China), Dulbecco's Modified Eagle Medium (DMEM), Fetal Bovine Serum (Hyclone, America), Trypsin (Gibco, America), TRizol, PrimeScript RT reagent kit, SYBR Green mix (CWBIO, China) were used.

### Immunohistochemistry

Gingival tissues were formalin-fixed, paraffin-embedded, sliced continuously, and dewaxed by xylene and ethyl alcohol. Antigen retrieval was performed by treatment with citric acid buffer heated in a water bath for 25 min. Next, the sections were treated with 3% H_2_O_2_ to block endogenous peroxidases, blocked for 1 h at room temperature (RT) with 10% blocking serum in Tris-buffered saline Tween-20 (TBST), and incubated with antibodies for leptin, OPG, and RANKL (all diluted 1:100) at 4 °C in a moist chamber overnight. The next day, slides were incubated with horse-radish peroxidase (HRP) -conjugated secondary antibodies at RT for 1 h. DAB was used for color development and hematoxylin was used for counterstaining. In the negative controls, the primary antibody was replaced with phosphate-buffered saline (PBS).

### Quantification of immunohistochemistry

Five views of each slice were randomly selected at high magnification (400×), and used to determine the immunoreactive score (IRS) 9 as follows. First, the staining intensity (SI) of each slide was assessed by two independent pathologists and assigned a number from 0 to 3 (0, no staining; 1, light yellow, 2, pale brown, and 3, dark brown). Second, a positive-cell-proportion score (PP) was evaluated (0 to 4) based on the percentage of positive cells (0, no positive cells; 1, <10% positive cells; 2, 10%-50% positive cells; 3, 50%-80% positive cells; and 4, >80% positive cells). The IRS for each slide was calculated by multiplying the SI and PP values. To eliminate the influence of subjective factors, the two researchers determined these scores independent of each other, and clinicopathological information of the sections was not disclosed to them in advance.

### Culture and identification of HGFs

HGFs were isolated from the gingiva of patients who underwent a third molar extraction and were cultured using the tissue-explant method. Tissue origin was determined by immunocytochemical staining for vimentin and keratin.

Fourth- generation HGFs under favorable growth conditions were inoculated in 6-well plates at a density of 1×10^5^ cells/well, and 2 mL DMEM was added to each well. Cells were left to grow under favorable conditions until they reached 100% confluency. Leptin was then added at various concentrations (0, 0.1, 0.5, 1, 5, or 10 µg/mL), and the cells were collected after 24 h with trypsin.

### RNA isolation, reverse transcription, and quantitative real-time PCR

Total RNA extraction was accomplished using the Trizol method add 1 mL Trizol for 15 min, then add 0.5 mL trichloromethane, oscillate for 15 s, and stand at RT for 10 min. Centrifugation at 4 °C, 12000×*g* for 15 min, isolate supernatant; add 0.5 mL isopropanol, and stand at RT for 10 min. Centrifugation at 4 °C, 12000×*g* for 10 min, discard the supernatant; add 1 mL 75% ethanol and gently wash the precipitate, discard the supernatant, followed by airing). The relative mRNA levels of *OPG* and *RANKL* were determined by RT-qPCR using the 2^-ΔΔCT^ method with *GAPDH* as the internal reference gene. The above steps (from HGF inoculation to RT-qPCR) were repeated three times, and three wells were processed per concentration group. The primers for the target genes and the internal reference gene are shown in Table 2.

### Statistical analysis

IBM SPSS Statistics 24.0 (IBM Corp, Armonk, NY, USA) was used for statistical analysis. All data are presented as mean ± SD. To evaluate the differences in IRS, one-way analysis of variance (ANOVA) was used for two-group comparisons and Tukey's test was used for multiple-group comparisons. Pearson correlation analysis was used to analyze the correlation between leptin and each of the other two proteins, OPG and RANKL. In the case of qPCR results, single-factor *t*-tests were used to evaluate the differences between groups. In all analyses, *p* < 0.05 was considered statistically significant.

## Results

### Leptin expression decreases with the progression of inflammation

**Figure 1:**
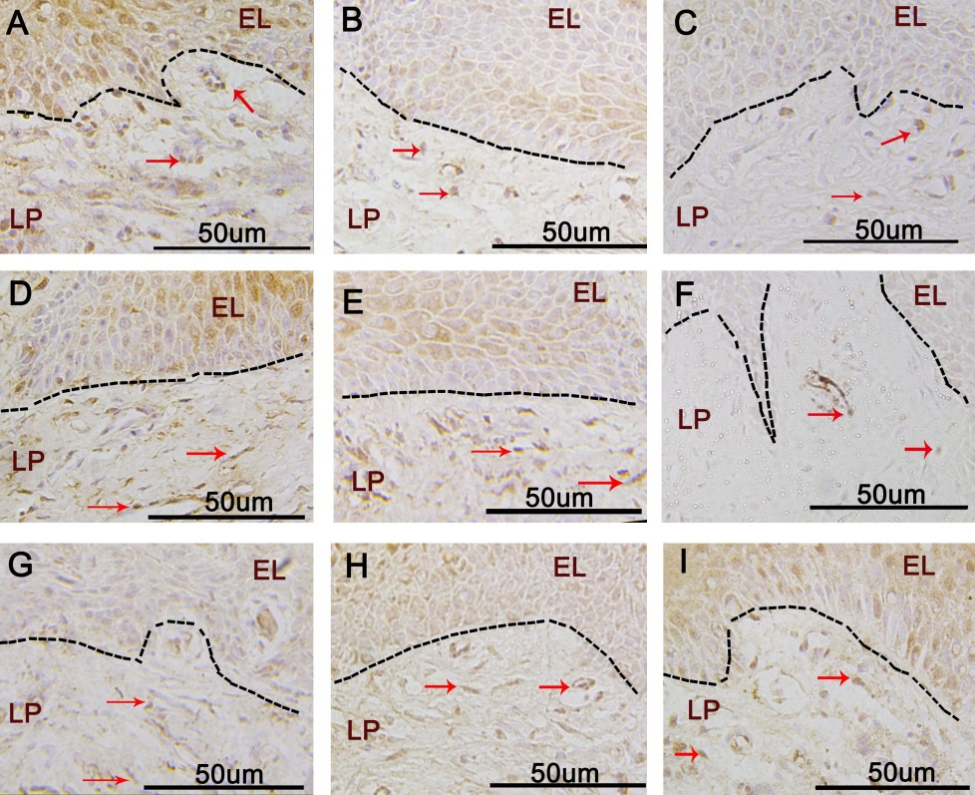
Immunohistochemical staining of Leptin, OPG, and RANKL in control and chronic periodontitis groups. **A-C.** Immunohistochemical staining of Leptin in the control group(A), the moderate chronic periodontitis group (B), and the severe chronic periodontitis group (C). **D-F.** Immunohistochemical staining of OPG in the control group (D), the moderate chronic periodontitis group (E), and the severe chronic periodontitis group (F). **G-I.** Immunohistochemical staining of RANKL in the control group (G), the moderate chronic periodontitis group (H), and the severe chronic periodontitis group (I). Red arrows indicate the positive cells. EL: epithelial layer, LP: lamina propria.

The expression of leptin in samples from the three groups (control, moderate chronic periodontitis, and severe chronic periodontitis) is shown in Fig. 1A-C. In all samples, leptin was located in the cytoplasm of gingival epithelial cells and the inflammatory cells in the connective tissue. In the control group, leptin was widely expressed in the lamina propria with deep staining (Fig. 1A). The expression levels decreased with the progression of inflammation (Fig. 1B-C). The IRSs for leptin for the three groups are shown in Table 3. The differences among the three groups were statistically significant (*p* < 0.05).

### OPG expression decreases with the progression of inflammation

The samples from the three groups under investigation showed OPG mainly expressed in the cytoplasm of the gingival epithelial cells and the connective tissue (Fig. 1D-F). In the control and moderate chronic periodontitis groups, OPG was widely expressed in the lamina propria (Fig. 1D-E), whereas the expression level was considerably lower in the severe chronic periodontitis patient samples (Fig. 1F). The IRSs of the three groups are shown in Table 4. There were statistically significant differences between the control and the severe chronic periodontitis groups, as well as between the moderate and the severe chronic periodontitis groups (*p* < 0.05). In contrast, the difference between the control and the moderate chronic periodontitis groups was not statistically significant (*p* > 0.05).

### RANKL expression increases with the progression of inflammation

RANKL expression was mainly found in the cytoplasm of gingival epithelium and the lamina propria (Fig. 1G-I). There was little expression of RANKL in the gingival epithelial cells and connective tissue of the control group (Fig. 1G); however, the expression levels increased with the progression of inflammation (Fig. 1H-I). The IRSs of the three groups are shown in Table 5; there were significant differences in the expression of RANKL among them (*p* < 0.05).

### Leptin levels are positively correlated with OPG and negatively correlated with RANKL

The correlation coefficient between leptin and OPG was r = 0.905, suggesting a positive correlation between the expression levels of these two proteins in the gingiva. In contrast, the protein expression levels of leptin and RANKL in the gingiva were negatively correlated (r = -0.635). In both cases, the correlation was statistically significant (*p* < 0.01) (Figure 1).

### Correlations between staining intensities of leptin, OPG, and RANKL and clinical indices

The staining intensities of leptin, OPG, and RANKL were correlated with the periodontal clinical index (Table 6). The protein expression levels of leptin and OPG in the gingiva were negatively correlated with the periodontal clinical index, whereas RANKL was positively correlated with the periodontal clinical index.

### Identification of tissue-explanted cells as HGFs

**Figure 2:**
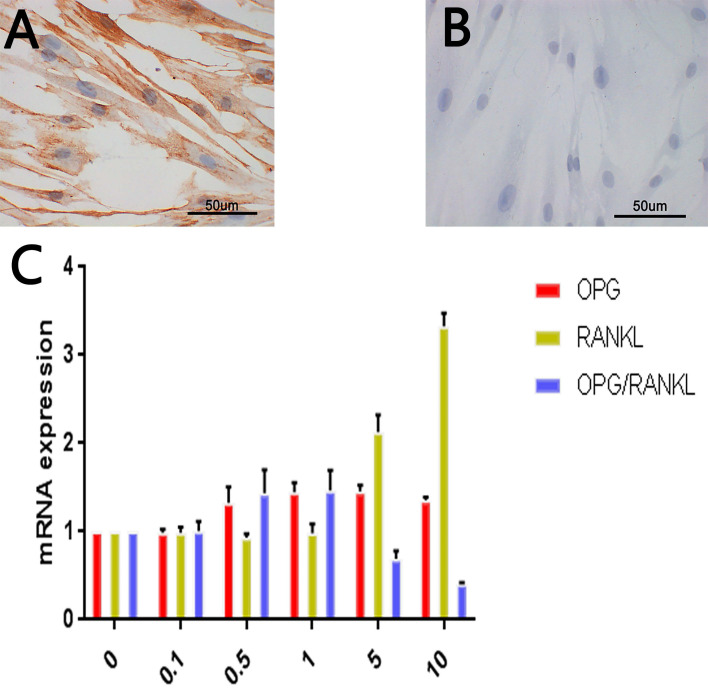
***RANKL* and *OPG* expression levels in leptin-treated HGF. A.** Immunohistochemical staining of vimentin in HGF. **B.** Immunohistochemical staining of keratin in HGF. **C.** Expression of *OPG, RANKL and OPG/RANKL* at mRNA level in different leptin concentration groups (ug/ml).

The spindle-shaped cells were positively stained for vimentin (Fig. 2A) and negatively stained for keratin (Fig. 2B). These results are consistent with the biological characteristics of mesoderm-derived cells and not epithelial cells, and, combined with the sampling site, led us to the conclusion that the cells were gingival fibroblasts.

### *RANKL* and *OPG* expression levels in leptin-treated HGF cultures

As shown in Fig. 2C, increasing leptin concentrations up to 5 µg/mL resulted in stronger *OPG* upregulation, with the *OPG* mRNA levels in the 5 µg/mL leptin group being 1.5 times higher than those in the control group (*p <* 0.05). However, upregulation at the highest leptin concentration (10 µg/mL) was slightly weaker than that at 5 µg/mL.

For *RANKL*, leptin at the three lowest concentrations (0.1, 0.5, and 1 µg/mL) did not significantly alter mRNA levels. However, treatment with the two highest concentrations (5 and 10 µg/mL) had a clear upregulatory effect. The maximum increase was observed using 10 µg/ml of leptin and resulted in *RANKL* expression 3 times greater than that of the control group (*p* < 0.05).

The *OPG/RANKL* mRNA ratio increased with leptin concentrations up to 1 µg/mL, with a corresponding ratio that was 1.5 times higher than that in the control group. However, a further increase in the leptin concentration led to a decrease in the ratio. At 10 µg/mL of leptin, the ratio was only 40% compared to that in the control group (*p* < 0.05).

## Discussion

Alveolar bone resorption is an important pathological manifestation of chronic periodontitis, and recent studies have shown that leptin is involved in the regulation of bone metabolism 10. *In vitro* studies have shown that, under leptin stimulation, the mRNA level of *OPG* increased and that of *RANKL* decreased significantly in both human peripheral blood mononuclear cells and mouse osteoblasts 11,12. Leptin inhibited adipocyte differentiation in human marrow stromal cells and promoted osteoblast differentiation 13. This study suggests that the regulation of OPG/RANKL pathway by leptin may be related to the differentiation of osteogenic precursor cells 14. In addition, leptin may promote RANKL expression through OB-rb/ERK and OB-rb/PI3K/AKT signaling pathways 15. In recent years, the OPG/RANKL pathway has been found to play an important role in periodontitis, as mice that over-express RANK exhibited severe alveolar bone loss 16, and periodontal therapy has been shown to reduce the level of RANKL in crevicular fluid, and therefore, decrease the RANKL/OPG ratio 17.

Leptin has been shown to be involved in a variety of bone metabolic diseases. Subcutaneous administration of leptin to rats with prednisolone-induced osteoporosis, a condition characterized by decreased serum OPG and increased serum RANKL levels, counteracted the effects of prednisone on OPG and RANKL levels and improved bone mineral density and content 18. Simopoulou *et al.* found that leptin and its receptor mRNA and protein are expressed in osteoarthritis articular chondrocytes, and found higher mRNA expression levels in advanced osteoarthritis cartilage compared to minimal osteoarthritis; they thought it may be related to the grade of cartilage destruction, indicating that leptin can indeed affect cartilage metabolism directly 19.

Previous studies 4-6 have shown that leptin may be involved in the development of chronic periodontitis. In the present study, leptin expression in the gingival tissue of the control group was significantly higher than that in the two chronic periodontitis groups. Moreover, the severe group had lower leptin levels than the moderate group. This is consistent with the results of previous studies employing the enzyme-linked immunosorbent assay (ELISA) 20. RANKL expression showed the opposite trend: while it was weak in the control group, it increased significantly with the development of chronic periodontitis. In the case of OPG, the control group had a significantly higher level than the severe group; however, no significant difference was detected in OPG expression between the control group and the moderate chronic periodontitis group. Our results can be considered consistent with those of previous studies 21,22, although they, unlike us, observed significant differences in OPG levels between the control and the moderate chronic periodontitis groups. This minor discrepancy may be attributed to differences in the ethnicity of the subjects or to different experimental conditions. Further studies with larger sample sizes are required to explore this fully. In addition, results of the correlation analysis of leptin, OPG, and RANKL with the periodontal clinical indicators was also consistent with the previously reported results 6,22.

Our *in vitro* experiments showed that *OPG* mRNA levels gradually increased with increasing leptin concentrations; *RANKL* showed no change with low leptin concentrations (0.1, 0.5, and 1 µg/mL) and increased at high concentrations (5 and 10 µg/mL); and lastly, the ratio of *OPG/RANKL* increased at low leptin concentrations and decreased at high concentrations. These results suggest that leptin may be involved in the progression of chronic periodontitis by regulating the OPG/RANKL system and thus altering the microenvironment of osteoclast differentiation in alveolar bone, something that has been observed in other bone metabolism-related diseases. The bone- destructive effect of leptin at high concentrations was previously suggested by Kim GS *et al*.^23^, who showed that local high concentrations of leptin had toxic effects on human bone marrow mesenchymal stem cells by the reduction of alkaline phosphatase activity and osteocalcin production in a dose-dependent manner. In the present study, we found that OPG expression increased gradually while the leptin concentration was increased at low concentrations. However, treatment with leptin at high concentrations lead to the increased RANKL expression level and decreased OPG/RANKL mRNA ratio. Notably, the results of our *in vitro* experiments were not fully consistent with our previous immunohistochemical results. The r-value of -0.635 for RANKL and leptin suggests there may be a negative correlation between RANKL and leptin. However, in *in vitro* experiments, the effect on RANKL only occurred at a higher leptin concentration, had a clear upregulatory effect. Therefore it suggests the RANKL expression level in gingival tissues may be regulated not only by leptin but also by other related factors, such as IL-6, TNF-α, and IL-17A, which could promote RANKL expression 24-26.

Leptin is a hormone secreted by adipocytes, and it is known as the paracrine factor released upon obesity and metabolic syndrome 27,28. Leptin has been found to modulate the innate and adaptive immunity responses in mucosal surfaces 29. The results of the present study indicate that leptin released under systemic conditions may dysregulate the innate immunity in the gingival epithelium and lamina propria, and then lead to the chronic periodontitis.

Zheng *et al*. 30 established a periodontal defect model in rats with osteoporosis in which rat bone marrow mesenchymal stem cells were transfected with adenovirus containing the human leptin gene; these were later implanted into the periodontal defect area. Well-formed bone tissue emerged, indicating the potential applications of leptin, both for periodontal regeneration in patients with osteoporosis, and as a potential new treatment strategy for chronic periodontitis.

In summary, this study investigated the expression of leptin, OPG, and RANKL in the gingival tissue of patients with chronic periodontitis, as well as the regulatory effect of leptin on the expression of OPG and RANKL in human gingival fibroblasts. Our results strongly suggest that leptin may be involved in the development of chronic periodontitis by modulating the OPG/RANKL system; however, further studies are required to fully explore the specific mechanisms through which leptin regulates chronic periodontitis.

## Figures and Tables

**Table 1:** Comparison of basic clinical data of three groups

Items	Normal	Moderate	Severe	*P*
Gender (male/female)	7/5	5/5	5/7	0.736
Age	29.58±3.50	31.50±2.91	31.42±3.39	0.301
BI	0.58±0.51	2.50±0.53	4.08±0.51	<0.001
PD	1.50±0.52	5.20±0.42	7.83±0.94	<0.001
CAL	0.04±0.14	3.40±0.52	5.33±0.49	<0.001

**Table 2:** The primers in real-time PCR

Genes	Primer sequence (5' → 3')
OPG	F: GCATTCTTCAGGTTTGCTG
	R: TGTGTTGCCGTTTTATCCT
RANKL	F: TCCATGCTCTTGACCTTGT
	R: ACCCCGTAATTGCTCCA
GAPDH	F: CCTTCCGTGTCCCCACT
	R: GCCTGCTTCACCACCTTC

**Table 3:** Immunoreactive score of leptin in different groups

Group	IRS	*P*			Variance analysis	
		Normal	Moderate	Severe	F	*P*
Control	7.75±3.26	-	0.032	<0.001	23.47	<0.05
Moderate	5.30±1.23	0.032	-	0.002		
Severe	1.75±1.09	<0.001	0.002	-		

Statistically significant:* p*<0.05.

**Table 4:** Immunoreactive score of OPG in different groups

Group	IRS	*P*			Variance analysis	
		Normal	Moderate	Severe	F	*P*
Control	7.25±3.30	-	0.134	<0.001	17.65	<0.05
Moderate	5.42±1.20	0.134	-	0.003		
Severe	2.08±1.03	<0.001	0.003	-		

Statistically significant:* p*<0.05.

**Table 5:** Immunoreactive score of RANKL in different groups

Group	IRS	*P*			Variance analysis	
		Normal	Moderate	Severe	F	*P*
Control	3.50±1.10	-	0.003	<0.001	56.59	<0.05
Moderate	5.70±1.43	0.003	-	0.001		
Severe	9.70±1.73	<0.001	0.001	-		

Statistically significant:* p*<0.05.

**Table 6:** The correlations between the staining intensity of leptin, OPG and RANKL and clinical indexes

Periodontal clinical index	*r*			*P*
	leptin	OPG	RANKL	
PD	-0.722	-0.683	0.866	<0.01
CAL	-0.738	-0.685	0.857	<0.01
BI	-0.694	-0.672	0.883	<0.01

Statistically significant:* p*<0.01.

## Abbreviations

HGFs: Human gingival fibroblasts
BI: bleeding index
PD: probing depth
CAL: clinic attachment loss
OPG: Osteoprotegerin
RANKL: Receptor Activator for Nuclear Factor-κB Ligand
ELISA: enzyme-linked immunosorbent assay
IL-6: interleukin-6
TNF-α: tumor necrosis factor α
ERK: Extracellular Signal Regulated Kinase
OB-rb: leptin receptor
PI3K: Phosphatidylinositide 3-kinases
AKT (PKB): protein kinase B
